# A damage-aware NGS workflow for conservative species identification from ultra-degraded DNA

**DOI:** 10.1007/s00216-026-06606-y

**Published:** 2026-06-13

**Authors:** Stefania Morelli, Sara Romano, Giulia Cosenza, Sergio Abate, Livia Lombardi, Elena Pilli

**Affiliations:** 1 IRIS (Infrastruttura per la Ricerca e l’Identificazione degli Scheletri senza nome) Dipartimento di Biologia, Università degli Studi di Firenze, Florence, Italy; 2 Forensic Microanalysis Unit, Carabinieri Scientific Investigation Department of Rome, 00191 Rome, Italy

**Keywords:** Next-generation sequencing, Species identification, Degraded DNA, Forensic genomics, Taxonomic classification

## Abstract

Species identification from highly degraded DNA remains a major challenge across ecology, conservation genetics, wildlife forensics, and museum science, where samples are often scarce, contaminated, and embedded in complex matrices. Under these conditions, standard reference-based and metagenomic classifiers are prone to false-positive assignments, particularly when ultra-fragmented DNA and conserved genomic regions are not explicitly accounted for. Here, we present a damage-aware next-generation sequencing (NGS) workflow for conservative species identification from minute quantities of highly degraded DNA, designed to minimize misclassification in low-input and damage-rich datasets. The workflow integrates micro-sampling, half-uracil–DNA–glycosylase (half-UDG) library preparation, PCR duplicate removal, multi-genome mapping against a curated reference panel, and a post-mapping read-ubiquity classifier that distinguishes species-specific reads from those shared across conserved loci. Using collagen-rich substrates as a proof-of-concept, we demonstrated accurate species attribution from samples as small as 1 mm^2^, including mixtures and mineral-containing matrices. The workflow reliably identifies dominant biological sources, reduces false-positive assignments driven by conserved genomic regions, and remains robust to common physical and chemical treatments such as swelling, heating, and plaster addition. Overall, this study provides a proof-of-concept framework for conservative species identification in challenging degraded DNA contexts. The workflow may be adaptable to a broader range of degraded DNA contexts—including wildlife monitoring, regulatory enforcement, forensic investigations, and the analysis of processed biological materials—although further validation across diverse matrices will be required.

**Graphical Abstract:**

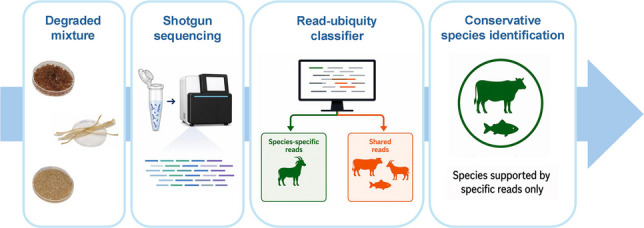

**Supplementary Information:**

The online version contains supplementary material available at 10.1007/s00216-026-06606-y.

## Introduction

Biological materials of animal and plant origin have been extensively used for millennia as pigments, dyes, binders, and adhesives in artworks and manufactured objects [1, 2]. Derived from natural or agricultural sources, these materials were selected for their extractability and functional properties, contributing to both aesthetic quality and long-term durability [3]. Among them, animal glues—rich in collagen—have played a central role not only as binders for pigments [4] but also in ground layers, gilding, and architectural decoration [5–10]. Produced by boiling skins, bones, tendons, or fish swim bladders, animal glues encompass a wide range of products with distinct mechanical and adhesive properties (Table 1).

**Table 1:** Main types of animal glues mentioned in technical treatises and available on the market, along with their commercial names, origin, properties, and main applications

Commercial name	Origins	Properties	Main applications
Bone glue	Mammalian bones, primarily	Low-refined glue; low collagen content; poor adhesive properties	Rarely used in cultural heritage
Hide glue	Scraps of skins and cartilaginous tissues, primarily bovine	Referred to as “colla di spicchi o di caravella” in ancient texts; commonly known as “strong glue”; high adhesive properties	Adhesive, painting binder, binder for preparatory layers, consolidant (restoration)
Parchment glue	Parchment scraps	Highly refined gelatin, intended for specific uses	Painting binder (miniature)
Rabbit hide glue	Scraps of rabbit skins and tissues, or other animals (sheep and goat)	Good adhesive and cohesive power, suitable as a binder	Painting binder, binder for preparatory layers, consolidant (restoration), binder for stuccoes
Fish glue	Fish skins and bones	Lower structural stability compared to others, limited use in cultural heritage	Fixative
Sturgeon glue (Isinglass)	Sturgeon swim bladders	Highly refined and valuable glue	Consolidant (restoration)

Their biological origin often reflects historical production practices, geographic availability of raw materials, and trade networks, making species attribution relevant to cultural heritage diagnostics, forensic attribution, and regulatory investigations.

Accurate identification of the biological origin of collagen-based materials remains challenging, particularly when only minute amounts of sample can be recovered. Traditional analytical approaches—including vibrational spectroscopy (Fourier transform infrared spectroscopy, near-infrared spectroscopy, Raman) [11], chromatographic techniques (gas chromatography–mass spectrometry and high-performance liquid chromatography), immunoassays [12–14], and chemiluminescence-based methods [15–17]—provide valuable information on chemical composition but often lack the taxonomic resolution required for reliable species-level identification. These limitations are especially pronounced in micro-sampled, mixed, or highly degraded matrices, where chemical similarity among collagen sources and low analyte abundance hinder discrimination [3, 18]. Comparable constraints have been reported across other disciplines dealing with degraded biomaterials, including conservation genetics, wildlife forensics, and biomedical research [19, 20].

DNA-based approaches inspired by ancient DNA research offer a powerful alternative, as they are specifically designed to recover and authenticate highly fragmented molecules while accounting for post-mortem damage patterns [21–23]. PCR-based assays have been sporadically applied to collagenous substrates [3, 24, 25], but their effectiveness is limited by primer bias, low template complexity, and an increased risk of false-positive assignments, particularly when closely related species share conserved genomic regions. Shotgun sequencing approaches overcome some of these constraints, yet species attribution from ultra-degraded DNA remains non-trivial. Widely used reference-based and metagenomic classifiers—such as Kraken2 [26], Centrifuge [27], and related tools—are primarily optimized for modern, high-quality DNA and often operate directly on raw FASTQ files. Similarly, tools such as FastQ Screen [28], used in ancient and paleogenomic studies for contamination screening [29], provide valuable multi-genome mapping but are not designed for conservative species attribution in damage-rich datasets, as they do not explicitly account for PCR duplication, post-mortem damage, or the ambiguity arising from conserved loci.

As a result, ultra-fragmented DNA embedded in complex matrices is particularly prone to misclassification, where short reads originating from conserved genomic regions are spuriously assigned to incorrect taxa. This issue has been highlighted by large-scale benchmarking studies of metagenomic classifiers and contamination analyses [30, 31], underscoring the need for workflows that explicitly prioritize reliability over maximal sensitivity when dealing with degraded material.

**Fig. 1:**
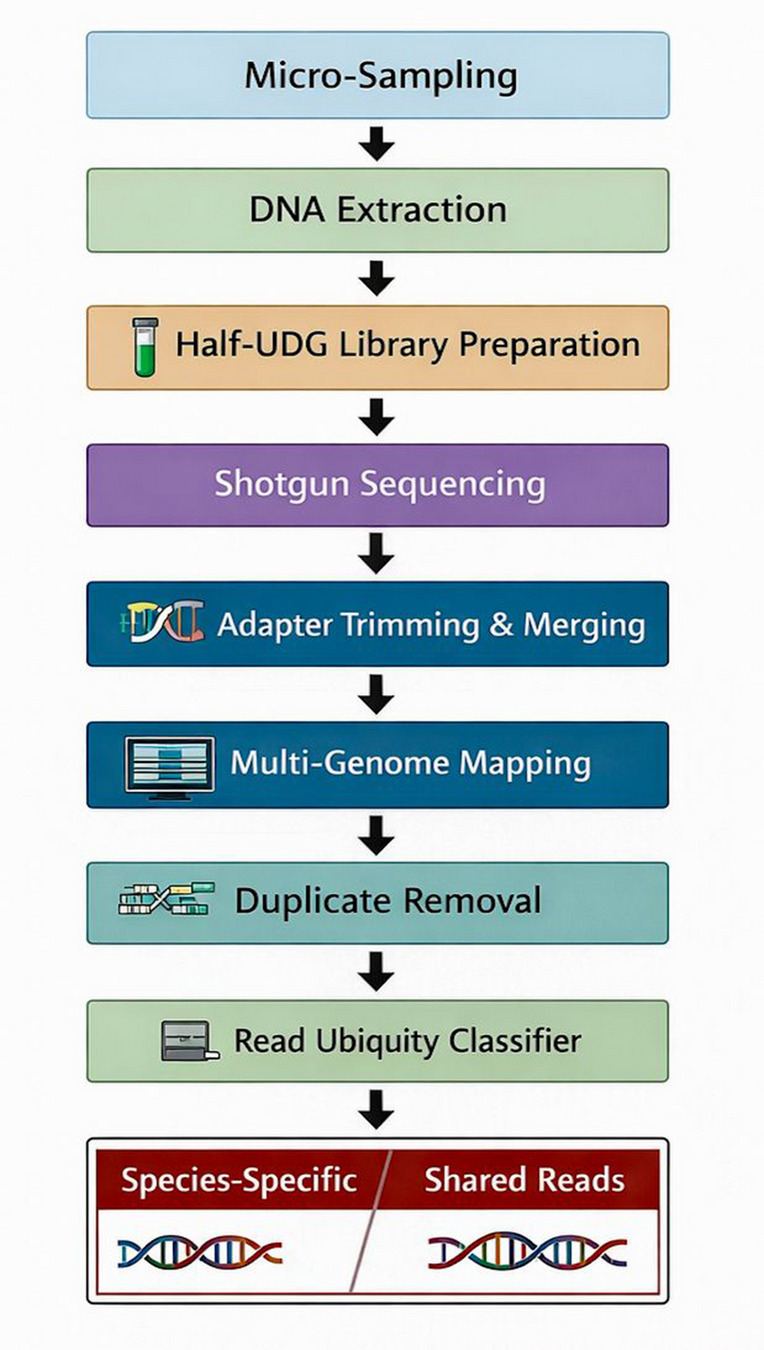
Schematic overview of the damage-aware NGS workflow used for conservative species identification from degraded DNA. The pipeline includes micro-sampling, DNA extraction, half-UDG library preparation, shotgun sequencing, read preprocessing, multi-genome mapping, duplicate removal, and classification of reads into species-specific and shared categories

Here, we introduce a minimally invasive, damage-aware next-generation sequencing (NGS) workflow for conservative species identification from highly degraded collagen-rich substrates. The main contribution of this study is the development of a conservative post-mapping classification strategy designed for damage-rich sequencing datasets, where ultra-short fragments and conserved genomic regions frequently cause misassignment. The workflow integrates (i) micro-sampling (~ 1 mm^2^), (ii) half-uracil–DNA–glycosylase (half-UDG) double-indexed library preparation, (iii) duplicate-aware processing, (iv) multi-genome mapping against a curated reference panel, and (v) a post-mapping read-ubiquity classifier that distinguishes species-specific reads from those shared across conserved genomic regions. DNA authenticity is assessed through damage-pattern analysis using established tools. We demonstrate the performance of this workflow using commercial animal glues and experimentally prepared mock-ups, including mineral-containing matrices that replicate historical preparation practices. Beyond this proof-of-concept, the approach may provide a useful methodological framework for species identification in damage-rich genomic datasets, with potential applications in cultural heritage science, wildlife monitoring, forensic investigations, regulatory enforcement, and the analysis of processed biological materials. A schematic overview of the workflow is shown in Fig. 1.

## Material and methods

### Sample preparation

Three commercial glues were acquired: “Hide Glue in Cubes” (B sample), “Rabbit Skin Glue” (C sample), and “Isinglass Glue” (S sample). Based on the production certificate and Table 1, the B sample was mainly composed of scraps of skins and cartilaginous tissues, primarily bovine, the C sample of scraps of rabbit skins and tissues, or other animals (sheep and goat), and the S sample of sturgeon swim bladders. To assess the feasibility of DNA analysis and authenticate the glue composition, samples were taken directly from the purchased products.

**Fig. 2:**
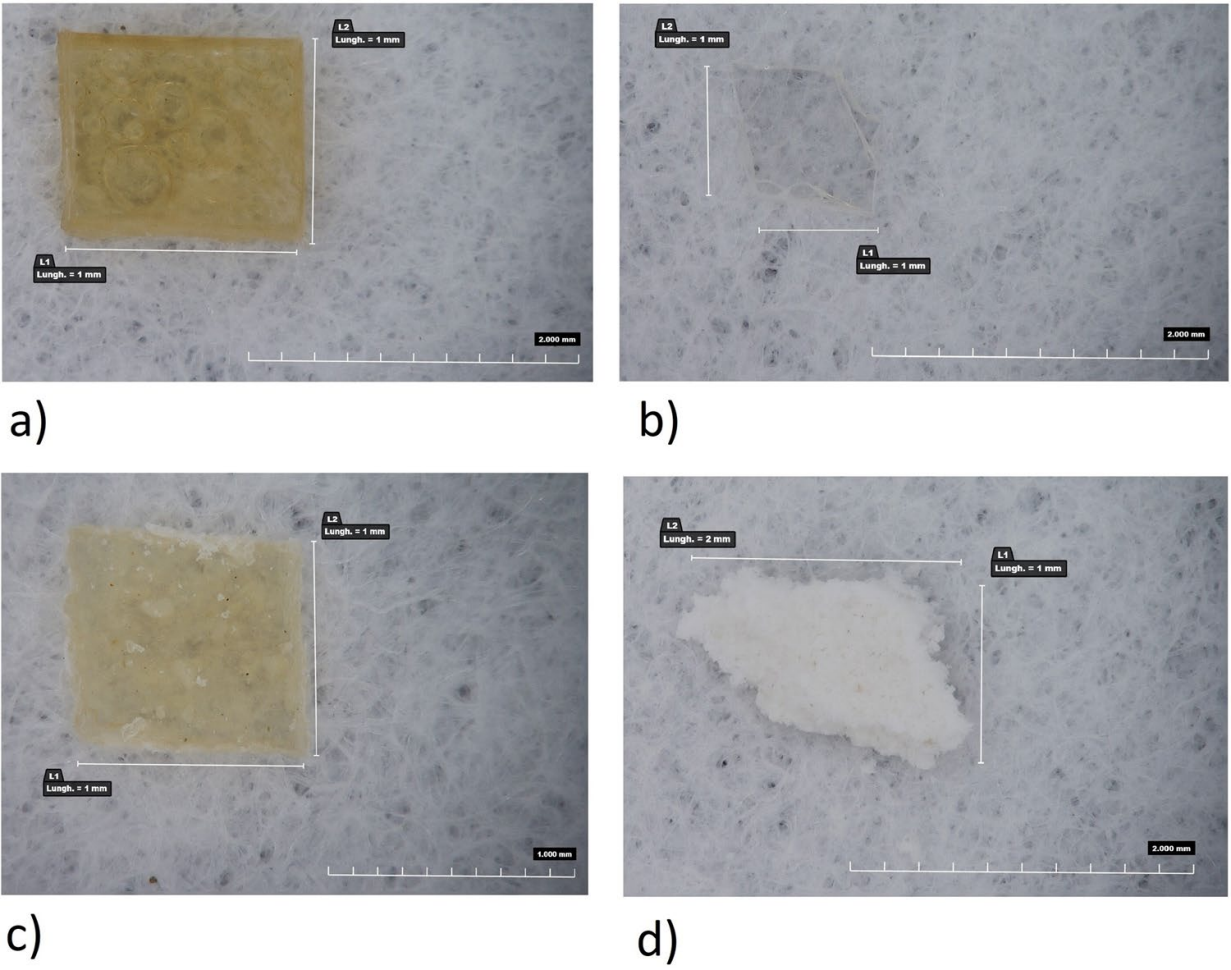
Specimen sections measuring 1 mm^2^ were excised from microscope slides for **a** pure Hide Glue in Cubes (B1); **b** pure Isinglass Glue (S1); **c** Hide Glue in Cubes and chalk mixture (BG1); and **d** Isinglass Glue and chalk mixture (SG1)

Based on the preliminary results of pure glue powder, mock-up samples were prepared by the Forensic Microanalysis Unit of the Carabinieri Scientific Investigation Department in Rome, to evaluate the method’s performance on complex matrices and its interaction with other painting components. This included glue spread on microscope slides and glue mixed with plaster, following medieval recipes. The swelling phase followed Cennini [32] and Marconi [33], involving overnight soaking in distilled water overnight at room temperature. For B, water was added to cover the grains; for S and C, a 1:7 glue-to-water ratio was used. The glue was then liquefied in a hot water bath maintained just below 100 °C to prevent protein degradation and replicate historical preparation conditions. Two specimens were prepared for each glue type: liquid glue spread on sterile microscope slides (B1 and S1), and glue mixed with sieved chalk (BG1 and SG1). The homogeneous mixture obtained was deposited on a sterile microscope slide. All mock-up samples were dried for approximately 18 h in a controlled environment to prevent contamination, then placed in an oven at 40–50 °C for 20 min. Using a scalpel, 1 mm^2^ sections were excised (Fig. 2) and stored in sterile vials.

### DNA extraction and quantification

All molecular analyses were conducted at IRIS, University of Florence. Samples were UV-radiated for 45 min using a Biolink DNA Crosslinker (Biometra) to remove any external contaminants. About 50 mg of powder was obtained from Hide Glue in Cubes (BA sample) and Rabbit Skin Glue (CA sample), and 30 mg from Isinglass Glue (SA sample). Granular glues (BA and CA) were pulverized with a mortar; stick glue (SA) was sampled using a low-speed rotary drill (Dremel® 300 series). The pulverized glue and mock-up samples were digested in 1 ml of extraction buffer (0.01 M Tris buffer, 0.01 (м) NaCl solution, 1% SDS, 0.5 mg/ml proteinase K, 10 mg/ml DTT, and 0.001 (м) PTB (*N*-phenacylthiazolium bromide) [34]) and incubated overnight at 55 °C. DNA was purified using the High Pure Viral Nucleic Acid Large Volume Kit (Roche), yielding 100 μl of eluted DNA in elution buffer (EB). DNA concentration was measured using the Qubit® dsDNA HS kit according to the user manual.

### Library preparation and sequencing

DNA extracts were subsequently converted into a double-stranded, dual-indexed Illumina DNA library following published protocols [35, 36]. A half uracil–DNA–glycosylase (half-UDG) treatment was applied to partially remove deaminated cytosines caused by DNA damage [37]. Libraries were quantified using Agilent 4150 TapeStation System and sequenced on the Illumina NovaSeq 6000 (Illumina, San Diego, CA, USA) platform (2 × 50 paired-end cycles). A shotgun approach was used, which yielded approximately twenty million reads per sample. Negative controls were included at all stages.

### Bioinformatic preprocessing

Raw reads were demultiplexed using barcode information. Subsequently, the demultiplexed data were analysed using pipelines dedicated to degraded samples. This step ensures that each DNA fragment is accurately identified and processed, minimizing potential errors and maximizing the quality of the data obtained. AdapterRemoval [38] was used to trim adapters and filter low-quality reads, retaining sequences > 30 bp and with quality scores ≥ 30. Paired-end reads were then merged with the Clip&Merge [39], with a minimum overlap of 10 bp required for successful merging.

### Reference panel and mapping strategy

The reference panel was curated to include species commonly used in glue production of collagen-based glues, as documented in historical and commercial sources, ensuring relevance to the investigated materials and enabling controlled taxonomic comparison within a defined reference framework. Reference genomes were downloaded from RefSeq online databases for species commonly used in glue production: *Bos taurus*, *Acipenser ruthenus*, *Oryctolagus cuniculus*, *Capra hircus*, *Equus caballus*, *Sus scrofa*, and *Ovis aries*. Additionally, *Homo sapiens* were included to account for potential contamination. *Salmo salar* was included to distinguish sturgeon DNA from other fish species. *Salmo salar* was selected among available fish genomes in the RefSeq database due to its classification within the same class as *Acipenser ruthenus* (Actinopterygii) but in a different order, making it a relevant comparator. All genomes were merged into a single reference sequence for multigenome alignment. Mapping was performed using the BWA aln [40]. To ensure high confidence alignments in the context of highly degraded DNA, only reads with a mapping quality (MAPQ) score ≥ 30 were retained for downstream analyses. Duplicate reads were removed using Dedup [39]. Reads were classified as species-specific only if they mapped uniquely to a single reference genome with high mapping quality, whereas reads mapping to multiple genomes or with ambiguous alignment were classified as shared. DNA damage patterns at the 5′ and 3′ ends were assessed using MapDamage 2.0 [41].

### Reads ubiquity classifier and conservative calls

To evaluate read distribution across genomes, FastQ files were extracted using Samtools [42], and analysed using Bash scripts. These scripts identified reads uniquely mapped to one genome and those shared across multiple genomes, using BAM files with contig-level associations. This classification distinguished species-specific (“pure”) reads from shared ones, improving taxonomic resolution and reducing misclassification due to conserved loci. The logic of the read-ubiquity classifier is summarized in the Electronic Supplementary Material (see Supplementary Method -pseudocode-), where the procedures used to identify species-specific (pure) and shared reads are explicitly defined to ensure methodological reproducibility. Bootstrap resampling was performed on species-specific (pure) read counts for each sample (BA, CA, SA) to assess the stability of taxonomic proportions. A multinomial bootstrap approach was applied, generating 10,000 simulated datasets based on the observed read proportions. Confidence intervals (95%) were estimated as the 2.5th and 97.5th percentiles of the bootstrap distributions.

## Results

### Pure glue powder (BA, CA, and SA samples)

To assess the feasibility of species identification from degraded glue matrices, three commercial samples were analysed using a dedicated NGS workflow. After adapter trimming and quality filtering, reads were merged and PCR duplicates removed. The final number of unique reads was 11,595,572 for BA, 9,669,827 for SA, and 7,275,317 for CA, with differences attributable to pooling imbalance.

#### Reads mapping and classification

**Fig. 3:**
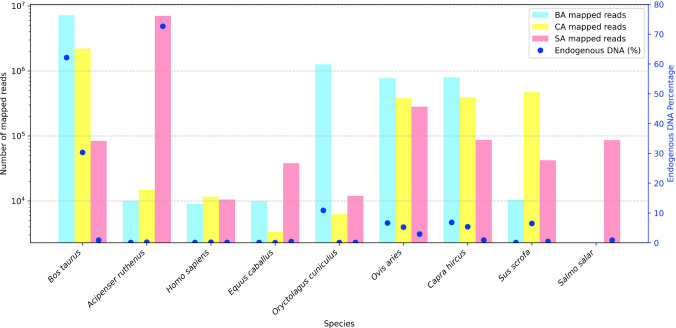
For each mapping analysis of the BA, CA, and SA samples, the number of reads mapped to each species (after duplicate removal) and the corresponding percentage of endogenous DNA are shown. Coloured bars represent mapped reads (light blue for BA, yellow for CA, pink for SA), while blue dots indicate the percentage of endogenous DNA. The graph uses dual *y*-axes: the left axis (in black) shows the number of mapped reads (log scale), and the right axis (in blue) shows the percentage of endogenous DNA

Mapping analysis revealed distinct species compositions across the three samples (BA, CA, SA) (Fig. 3).

In BA, *Bos taurus* was the dominant species, with over 7.2 million mapped reads and 62.14% endogenous DNA. *Oryctolagus cuniculus* accounted for 10.86%, while *Capra hircus* and *Ovis aries* showed similar endogenous percentages (6.83% and 6.65%, respectively). All other species, including *Equus caballus*, *Sus scrofa*, *Acipenser ruthenus*, and *Homo sapiens*, contributed less than 0.01% of mapped reads*.* In CA, *Bos taurus* remained the most represented species (30.31%), followed by *Sus scrofa* (6.47%), *Capra hircus* (5.37%), and *Ovis aries* (5.23%). *Oryctolagus cuniculus* was detected at low abundance (0.09%). SA was dominated by *Acipenser ruthenus*, with approximately seven million mapped reads and 72.66% endogenous DNA. All other species contributed (≤ 0.89%), with *Ovis aries* showing a slightly higher value (2.91%). Misincorporation patterns at the 5′ and 3′ ends of all samples were consistent with half-UDG treatment across all samples, indicating mild DNA degradation. Fragment length distributions were consistent across all samples, showing a peak around 50–55 bp, indicative of highly fragmented DNA typical of degraded materials (see Electronic Supplementary Material Fig. S1).

#### Reads ubiquity evaluation

The read-ubiquity classifier distinguished species-specific (pure) reads from reads shared across multiple reference genomes (see Electronic Supplementary Material Fig. S2). In BA, most reads were uniquely mapped, while a smaller fraction was shared across genomes. *Bos taurus* and *Acipenser ruthenus* showed the highest proportions of shared reads (36.88% and 32.02%, respectively). In contrast, *Sus scrofa*, *Equus caballus*, *Oryctolagus cuniculus*, and *Homo sapiens* showed high proportions of species-specific reads (80.5–88.9%).

The CA sample displayed a more heterogeneous profile. *Sus scrofa* exhibited the highest specificity (83.36%), while *Bos taurus* and *Acipenser ruthenus* showed lower species-specific proportions (42.28% and 44.90%). *Capra hircus* and *Ovis aries* exhibited intermediate specificity (approximately 55%).

In SA, *Acipenser ruthenus* showed high specificity, with 92.23% of reads classified as species-specific. Other species displayed lower specificity, with shared reads accounting for the majority of mapped sequences.

#### Reconstructing glue composition

**Fig. 4:**
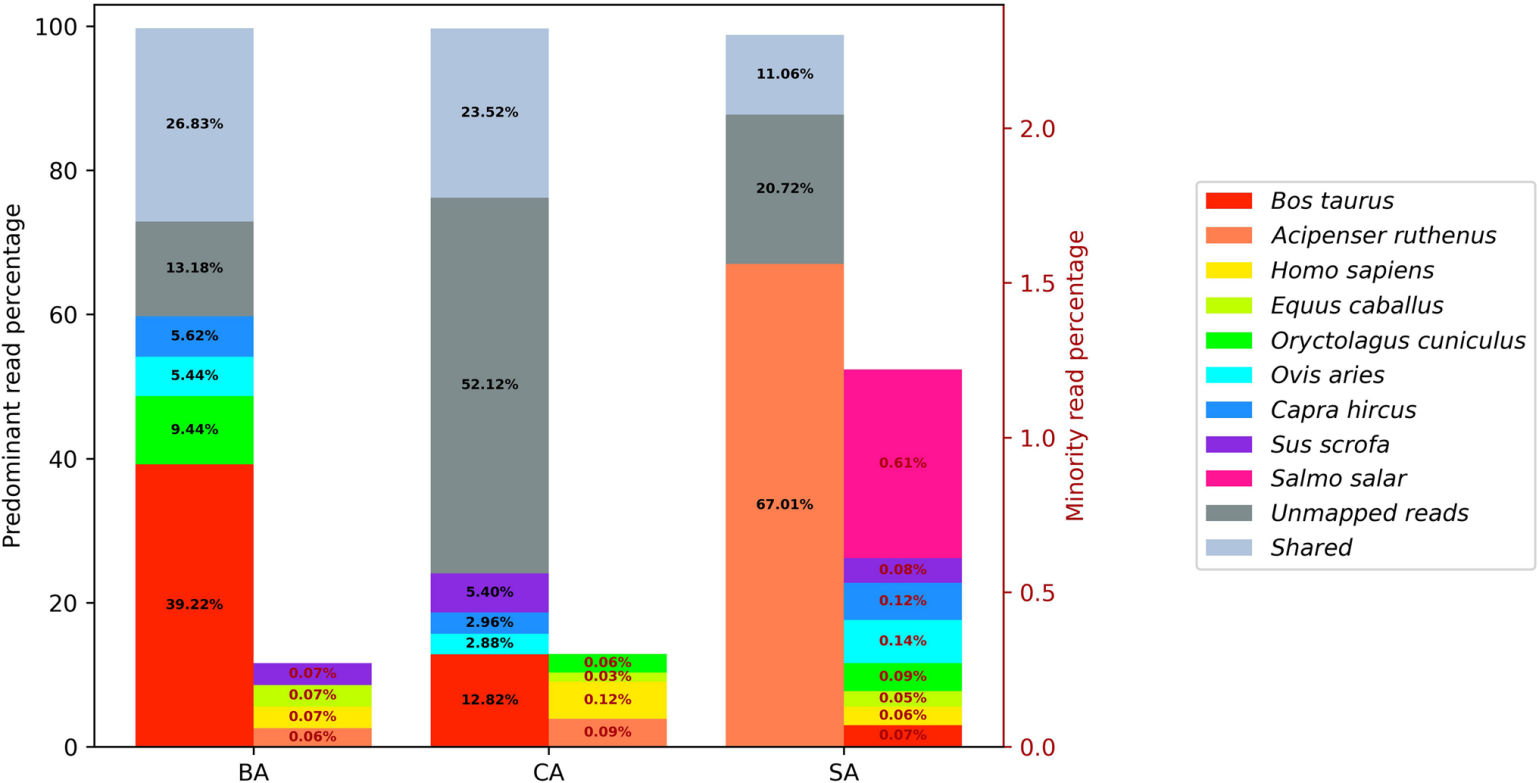
Percentages of the investigated components (*Oryctolagus cuniculus*, *Bos taurus*, *Acipenser ruthenus*, *Capra hircus*, *Equus caballus*, *Sus scrofa*, *Ovis aries*, *Homo sapiens*, and *Salmo salar*) in the BA, CA, and SA samples. The chart shows the relative proportions of each species based on the number of uniquely mapped reads (species-specific “pure” DNA). Each colour represents a distinct species. The light grey segment indicates reads that mapped to more than one species and could not be unambiguously assigned (shared reads), while the dark grey segment represents reads that did not map to any of the investigated species (unmapped reads). Each sample is represented by two adjacent bars (left and right), allowing the use of dual y-axes to better visualize both high and low percentages

Species composition and read classification (species-specific, shared, and unmapped) are shown in Fig. 4.

**Fig. 5:**
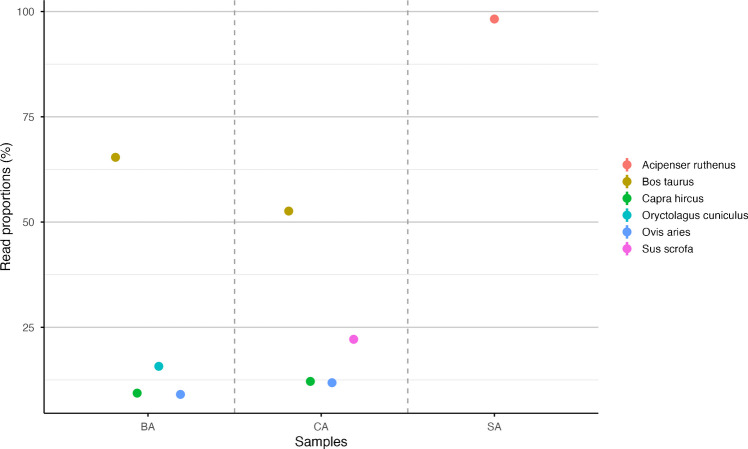
Bootstrap estimates of species-specific read proportions (mean ± 95% CI) for abundant taxa (> 1%) in samples BA, CA, and SA. Points represent mean proportions and error bars indicate 95% confidence intervals derived from multinomial bootstrap resampling (*n* = 10,000). Error bars are not visible at this scale due to the extremely narrow confidence intervals, reflecting the high stability of the estimates

BA was predominantly bovine with *Bos taurus* accounting for 39.22% of species-specific reads, while additional contributions from *Oryctolagus cuniculus*, *Ovis aries*, and *Capra hircus* were observed at lower proportions. CA exhibited a mixture with shared reads (23.52%) and unmapped reads (52.12%) accounting for the majority of sequences. Among the species-specific reads, *Bos taurus* was the most represented taxon (12.82%), followed by *Sus scrofa* (5.40%), *Capra hircus* (2.96%), and *Ovis aries* (2.88%), whereas *Oryctolagus cuniculus* contributed only minimally (0.09%). SA showed a distinct profile dominated by *Acipenser ruthenus* (67.01%), with only minor contributions from other taxa. Shared reads represented 26.83%, 23.52%, and 11.06% of mapped sequences in BA, CA and SA, respectively. Bootstrap analysis showed consistently narrow confidence intervals across all samples, indicating high stability of species-specific read proportions and confirming that the observed taxonomic patterns are not driven by stochastic variation (Fig. 5). Low-abundance species (< 1%) showed similarly narrow confidence intervals and are reported in Electronic Supplementary Material (Tables S1-S3).

### Mock-up samples (B1, BG1, S1, and SG1)

Mock-up samples prepared from B and S glues were analysed to assess performance on minute sample sizes and complex matrices. DNA was extracted and sequenced from 1 mm^2^ section of each mock-up sample. After preprocessing and duplicate removal, the number of unique reads was 14,670,543 for B1; 9,334,743 for BG1; 4,578,572 for S1; and 3,887,155 for SG1.

#### Reads mapping and classification

**Fig. 6:**
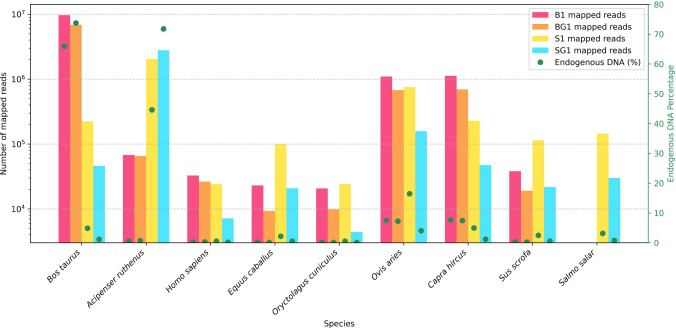
For each mapping analysis of the B1, BG1, S1, and SG1 samples, the number of reads mapped to each species (after duplicate removal) and the corresponding percentage of endogenous DNA are shown. Coloured bars represent mapped reads (pink for B1, orange for BG1, yellow for S1, and light blue for SG1), while green dots indicate the percentage of endogenous DNA. The graph uses dual *y*-axes: the left axis (in black) shows the number of mapped reads (log scale), and the right axis (in blue) shows the percentage of endogenous DNA

Mapping results for mock-up samples are shown in Fig. 6.

B1 and BG1 closely mirrored the composition of the BA sample, with *Bos taurus* showing the highest endogenous DNA percentages (65.95% in B1 and 73.73% in BG1). *Capra hircus* and *Ovis aries* followed in abundance with similar proportions (~ 7.4% and ~ 7.3%, respectively), while *Oryctolagus cuniculus* showed reduced endogenous DNA compared to BA. S1 and SG1 displayed profiles broadly consistent with SA, with *Acipenser ruthenus* as the dominant species (44.59% in S1 and 71.76% in SG1). However, S1 showed a more heterogeneous composition, with *Ovis aries* representing the main secondary component (16.46%), followed by smaller contributions from *Capra hircus*, and *Bos taurus*. Damage patterns in all mock-up samples were consistent with half-UDG treatment and comparable to those observed in the corresponding glue powders.

Comparable fragment length patterns were observed in both pure glue samples and mock-up materials, suggesting that processing conditions such as heating and matrix incorporation did not substantially alter DNA fragmentation profiles (see Electronic Supplementary Material Fig. S3). Minor irregularities in fragment length distributions were observed in SG1, likely reflecting stochastic variation rather than systematic differences in DNA degradation.

#### Reads ubiquity evaluation

The distribution of species-specific and shared reads was evaluated in all mock-up samples (see Electronic Supplementary Material Fig. S4). Each sample (B1, BG1, S1, SG1) displayed a combination of species-specific (“pure”) and shared reads, consistent with the patterns observed in the corresponding glue powders (BA and SA). Comparison between B1 and BG1 showed a slightly higher proportion of species-specific reads in B1 across most taxa, despite the lower total read count in BG1. Similarly, S1 and SG1 exhibited comparable distributions of pure and shared reads, with SG1 showing a higher proportion of uniquely mapped reads for most species, with the exception of *Salmo salar*. Overall, the distribution of reads across taxa remained consistent among mock-up samples, indicating that sample processing and matrix composition did not substantially alter read specificity.

#### Species composition of mock-up samples

**Fig. 7:**
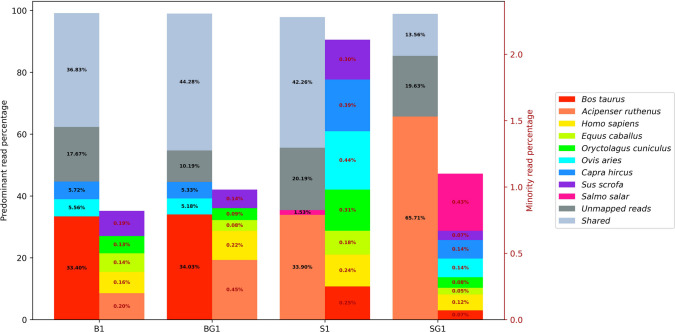
Taxonomic composition of samples B1, BG1, S1, and SG1 based on the combination of mapped reads and the percentage of species-specific (pure) DNA. Each colour represents a different investigated species (*Bos taurus*, *Capra hircus*, *Equus caballus*, *Oryctolagus cuniculus*, *Sus scrofa*, *Ovis aries*, *Acipenser ruthenus*, *Homo sapiens*, *Salmo salar*), while light grey indicates shared reads and dark grey represents unmapped reads. The graph highlights both predominant (on the left) and minority (on the right) components, allowing a comparative evaluation of taxonomic signals across the four mock-up samples

Species composition based on species-specific reads is shown in Fig. 7.

Each sample displayed a consistent taxonomic profile, with compositions closely resembling those of the corresponding reference glue powder (BA and SA). B1 and BG1 were dominated by *Bos taurus* (33.40% and 34.03%), accompanied by shared reads (36.83% in B1 and 44.28% in BG1). Minor contributions from *Capra hircus*, *Ovis aries*, and *Oryctolagus cuniculus* were detected. S1 and SG1 samples showed a comparable taxonomic profile to the SA sample. They were dominated by *Acipenser ruthenus* (33.90% and 65.71%), with shared reads accounting for 42.26% and 13.56%, respectively. Minor contributions from *Salmo salar* and other taxa were observed.

## Discussion

Reliable species identification from highly degraded DNA remains a critical methodological challenge in many genomic applications, particularly when working with low-input material, complex substrates, and closely related taxa. Under these conditions, standard reference-based and metagenomic classification approaches may generate false-positive assignments driven by ultra-short fragments, conserved genomic regions, and insufficient control of post-mortem damage and PCR duplication. The workflow presented here was specifically designed to address these issues by prioritizing conservative species calls over maximal sensitivity.

**Fig. 8:**
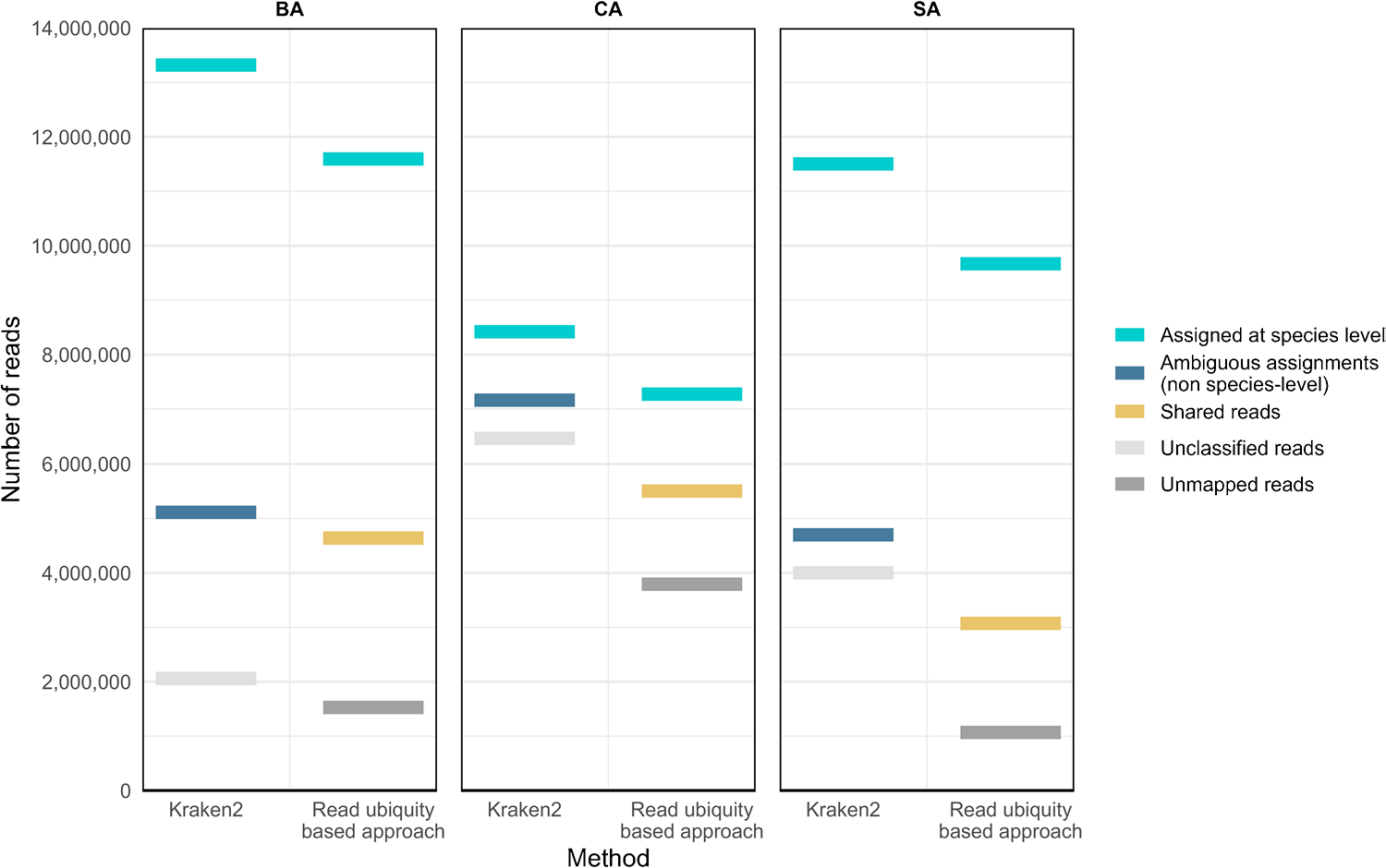
Comparison of read classification between Kraken2 and the read-ubiquity approach for samples BA, CA, and SA. In Kraken2, reads are reported as assigned at species level, ambiguous assignments (non-species-level), or unclassified. In the read-ubiquity approach, reads are reported as assigned at species level, shared, or unmapped

A central feature of this approach is the integration of damage-aware library preparation with a post-mapping read-ubiquity classifier. Unlike k-mer-based classifiers such as Kraken2 or Centrifuge, which operate directly on raw reads, our workflow evaluates taxonomic specificity after duplicate removal and multi-genome alignment, enabling conservative classification in highly degraded datasets. Our approach is complementary rather than alternative to metagenomic classifiers, as it prioritizes conservative species identification in contexts where false-positive assignments may have critical interpretative consequences. Rather than explicitly modelling contamination, the workflow handles potentially contaminant or ambiguous signals by classifying them as shared, thereby avoiding overinterpretation at the species level. To evaluate the performance of the proposed workflow, we compared its output with that obtained using a k-mer-based classifier (Kraken2) applied to the same datasets and reference panel (Fig. 8).

Kraken2 assigned a larger proportion of reads at the species level, whereas the read-ubiquity classifier resulted in a higher fraction of reads classified as shared or unmapped.

This difference reflects the underlying classification strategies: while k-mer-based approaches maximize sensitivity by assigning reads to the most specific possible taxonomic level, the read-ubiquity classifier adopts a more conservative approach, explicitly separating ambiguous reads that map to multiple genomes. This comparison is not intended as a comprehensive benchmarking exercise, as the performance of k-mer-based classifiers can be further improved through pipeline optimization and contamination-aware filtering.

As a result, the proposed workflow reduces the risk of overinterpretation of conserved genomic regions, which are particularly problematic in highly degraded DNA datasets.

This approach explicitly distinguished species-specific reads from those shared across reference genomes, thereby reducing misclassification risk among related taxa.

The application of the workflow to collagen-rich substrates demonstrates its robustness under conditions that are especially challenging for molecular identification. Collagen-based matrices are characterized by extensive DNA degradation, low endogenous content, and frequent mixtures of biological sources. Despite these constraints, species attribution remained consistent across pure materials and experimentally prepared mock-ups, including samples subjected to swelling, heating (up to 100 °C), and the incorporation of mineral components such as chalk. These results indicate that the workflow is resilient to common physical and chemical treatments that often accompany sample preparation in applied contexts. Overall, the consistency of fragment length distributions and taxonomic profiles confirms that the workflow operates effectively on short DNA fragments and supports its applicability to highly degraded DNA across different sample types.

The CA sample, labelled as rabbit skin glue, showed a marked discrepancy between the declared composition and the molecular signal. The very low proportion of *Oryctolagus cuniculus* and the higher contribution from *Bos taurus* indicated that the biological composition did not reflect the commercial label. In particular, the use of mixed raw materials during industrial processing represents a plausible explanation. Collagen-based glues are often produced from heterogeneous sources, and the reuse of processing equipment or the handling of multiple materials within the same production environment may result in the incorporation of DNA from different species. These observations highlighted the difficulty in distinguishing between the dominant biological source and the declared commercial label in highly processed materials. Additional analysis indicated that a substantial fraction of unmapped reads corresponds to bacterial DNA, suggesting the presence of non-target biological material and further supporting the complexity of the sample. Importantly, the read-ubiquity classifier does not aim to force unambiguous assignments when the available data do not support them. Instead, it provides a conservative framework in which ambiguous reads are explicitly classified as shared or uninformative. This design choice reflects a deliberate methodological stance: in damage-rich datasets, avoiding false-positive species calls is often more critical than maximizing taxonomic resolution. Such conservatism is particularly relevant in regulatory, forensic, and ecological applications, where erroneous species attribution may have legal, conservation, or interpretative consequences.

While the workflow is conceptually adaptable to other degraded DNA contexts, its performance outside collagen-based materials remains to be systematically evaluated. All components—from library preparation to post-mapping classification—are modular and may be adapted to other contexts involving degraded DNA, including wildlife monitoring, environmental samples, archaeological remains, and processed biological products. The approach is especially suitable when reference bias, shared genomic regions, and low template complexity limit the reliability of standard classification pipelines.

### Limitations

Several limitations should be acknowledged. While the present study focuses on collagen-based materials, the workflow is designed to be adaptable to other types of degraded samples. Its application to different matrices may therefore be feasible, although further validation across diverse substrates would be required to fully assess its transferability. The accuracy of species attribution depends on the completeness and phylogenetic coverage of the reference panel. Reads originating from unrepresented or closely related taxa may be preferentially assigned to the most similar available genome or classified as shared, potentially leading to biased or ambiguous taxonomic signals. This reflects a fundamental trade-off between sensitivity and specificity: a restricted reference panel improves interpretability and reduces spurious assignments but may limit the detection of unexpected or unrepresented species, whereas broader panels increase sensitivity at the cost of higher ambiguity.

In this study, the reference panel was intentionally restricted to species relevant to collagen-based glue production, enabling a controlled and application-specific comparison while prioritizing interpretability over exhaustive taxonomic coverage. More generally, reference databases should be carefully curated and tailored to the expected taxonomic composition of the samples.

Importantly, the read-ubiquity classifier does not force unambiguous assignment when evidence is insufficient, as reads mapping across multiple genomes are explicitly classified as shared. However, when the true source species is absent from the panel, species-specific assignments should be interpreted with caution.

Controlled mixtures with known species proportions would provide a more systematic framework to evaluate performance under varying levels of mixture complexity and cross-mapping and represent an important direction for future work. Although the dataset includes closely related taxa (e.g. bovine, ovine, and caprine species), further validation using controlled mixtures would be necessary to systematically assess the discriminatory power of the workflow.

The performance of the workflow may be influenced by fragment length, sequencing depth, and library complexity. While fragment length distributions are provided in this study, a systematic evaluation of detection thresholds across varying fragment sizes and coverage levels would represent an important direction for future work. Additionally, uneven sequencing depth or pooling imbalance may influence the absolute number of species-specific reads, although relative patterns remained stable across treatments in this study. Targeted capture strategies could enhance sensitivity in some contexts, but their effectiveness depends on prior knowledge of expected taxa and may be less suitable for unknown or heterogeneous samples.

## Conclusion

This study introduces a damage-aware, matrix-tolerant NGS workflow for conservative species identification from ultra-degraded DNA. By combining micro-sampling, half-UDG library preparation, duplicate-aware processing, multi-genome mapping, and a post-mapping read-ubiquity classifier, the workflow addresses key sources of misclassification that affect standard approaches in low-quality genomic datasets. Rather than maximizing species detection, the proposed framework prioritizes reliability and transparency in taxonomic assignment, explicitly distinguishing species-specific signal from ambiguity arising from conserved genomic regions. This conservative strategy is particularly well suited to applications in which false-positive species calls carry significant interpretative, regulatory, or legal consequences.

Application of the workflow to collagen-rich adhesives demonstrated its applicability within the tested substrates, with potential relevance for cultural heritage diagnostics, authentication studies, forensic attribution, and industrial anti-adulteration. The analysis revealed both concordance and discrepancies between declared and molecularly inferred species composition, while mock-up samples prepared using historical recipes—including mineral-containing matrices—yielded consistent species identification despite minimal input material. These results illustrate the robustness of the approach under realistic and operationally relevant conditions.

While some limitations remain, particularly regarding reference panel composition and sequencing depth, this workflow provides a proof-of-concept methodological framework for species identification in challenging, damage-rich genomic datasets.

Its conservative design supports more reliable interpretation of degraded DNA across ecological, forensic, regulatory, and applied sequencing contexts, offering a conservative complementary approach for degraded DNA classification when robustness and transparency are prioritized over maximal sensitivity.

## Supplementary Information

Below is the link to the electronic supplementary material.

Supplementary file1 (PDF 464 KB)

## Data Availability

The sequencing data generated in this study is available from the corresponding author upon reasonable request.
