# Supplementary material for: A damage-aware NGS workflow for conservative species identification from ultra-degraded DNA
Source: Anal Bioanal Chem. 2026 Jun 13;418(15):5021–32. doi: 10.1007/s00216-026-06606-y (PMC13388423; doi:10.1007/s00216-026-06606-y)
Supplement: Supplementary file 1 — Supplementary file1 (PDF 464 KB) [file 216_2026_6606_MOESM1_ESM.pdf]

Supplementary Information

A damage-aware NGS workflow for conservative species identification from ultra-degraded DNA

Analytical and Bioanalytical Chemestry

Stefania Morelli[1], Sara Romano[1], Giulia Cosenza[1], Sergio Abate[2], Livia Lombardi[2], Elena Pilli[1]

[1]IRIS (Infrastruttura per la Ricerca e l'identificazione degli Scheletri senza nome)

Dipartimento di Biologia Università degli Studi di Firenze, Italy.

[2]Forensic Microanalysis Unit - Carabinieri Scientific Investigation Department of Rome, 00191, Italy

Elena Pilli Corresponding author elena.pilli@unifi.it

Supplementary Method

Pseudocode

Algorithm 1 Counting total assigned reads per genome from a sorted BAM file and a contig-to-genome mapping file

```
Input: Sorted BAM file BAM_FILE; tab-delimited mapping file MAPPING_FILE containing pairs (contig, genome)
Initialize an empty associative dictionary genome_reads
For each pair (contig, genome) in MAPPING_FILE do
    Extract from BAM_FILE all alignments mapped to contig
    Remove secondary alignments
    Compute read_count as the number of unique read identifiers among the remaining reads mapped to the contig
    Initialize or update genome_reads[genome] by adding read_count
End for
For each genome in genome_reads do
    Write genome and genome_reads[genome] to the output file
End for
```

Algorithm 2 Counting species-specific (pure) read sequences per genome from a sorted BAM file and a contig-to-genome mapping file

```
Input: Sorted BAM file BAM_FILE; tab-delimited mapping file MAPPING_FILE containing pairs (contig, genome)
Extract all alignments from BAM_FILE and initialize empty associative dictionaries genome_sequences and pure_reads
For each alignment, store the contig name and the read sequence in a temporary data structure A
For each pair (contig, genome) in MAPPING_FILE do
    Extract from A the sequences associated with contig
    Sort the sequences and remove duplicate sequences for that contig
    Add these sequences to genome_sequences[genome]
End for
For each genome in genome_sequences do
    Collect all sequences associated with the other genomes
    For each sequence in genome_sequences[genome] do
        If the sequence is not present among the sequences associated with the other genomes then
            Increment pure_reads[genome]
        End if
    End for
End for
For each genome in pure_reads do
    Write genome and pure_reads[genome] to the output file
End for
```

Algorithm 3 Calculation of shared reads
Input: Output file from Algorithm 1 containing pairs (genome, total_reads); Output file from Algorithm 2 containing pairs (genome, pure_reads)
1: Initialize an empty dictionary shared_reads
2: For each genome in genome_reads do
3: total_reads = genome_reads[genome]
4: pure_reads = pure_reads[genome]
5: shared_reads[genome] = genome_reads[genome]− pure_reads[genome]
6: End for
7: For each genome in genome_reads do
8: Write genome and shared_reads[genome] to the output file
9: End For

Supplementary Figures

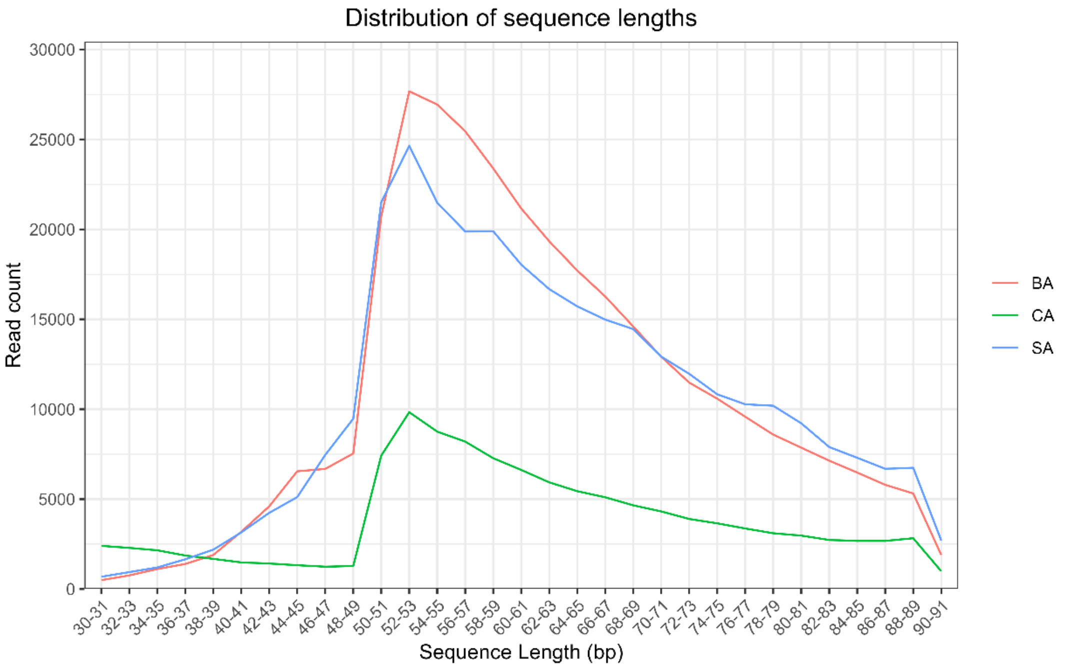

Fig. S1 Fragment length distribution of merged reads for pure glue samples (BA, CA, SA)

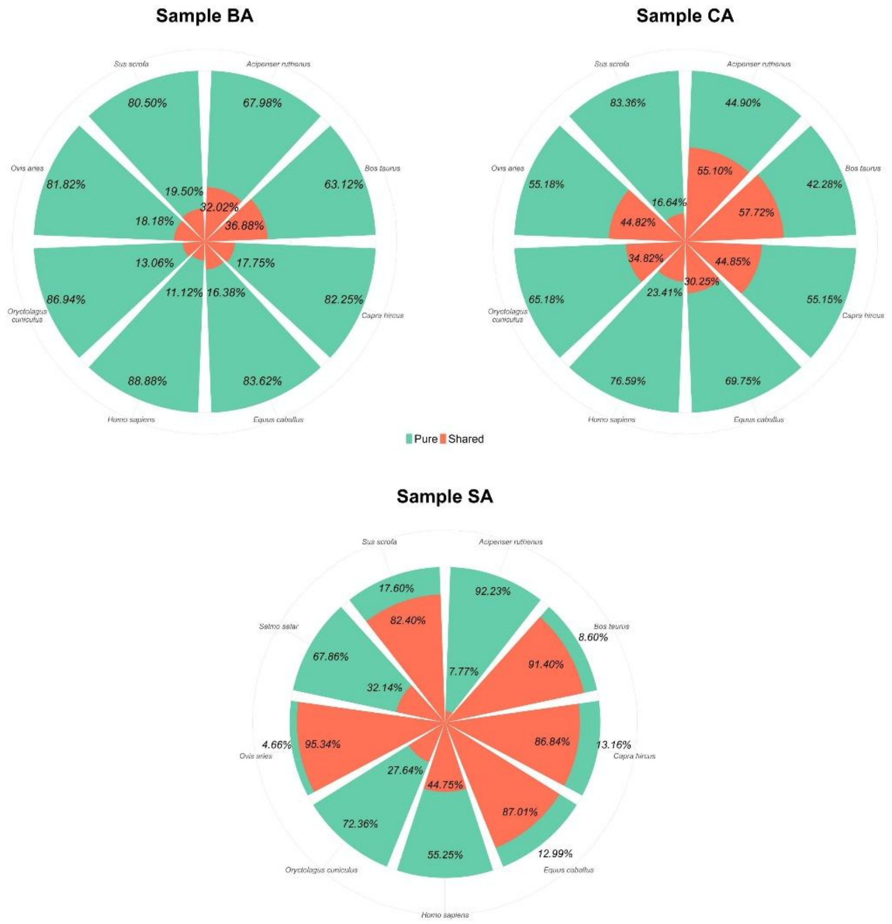

Fig. S2 Evaluation of the potential ubiquity of sequences mapped to the genomes of *Oryctolagus cuniculus, Bos taurus*, *Acipenser ruthenus, Capra hircus*, *Equus caballus*, *Sus scrofa*, *Ovis aries*, *Salmo salar*, and *Homo sapiens* for the BA, CA and SA samples. The percentage of reads unique to each species (pure) is shown in aquamarine green, while the percentage of reads shared among reference genomes (shared) is displayed in red

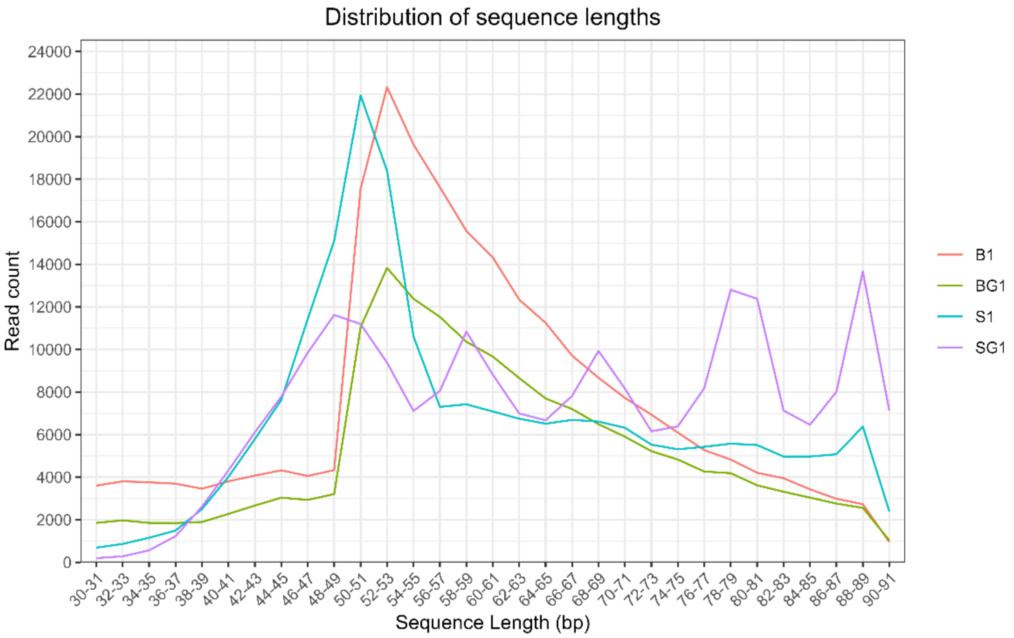

Fig. S3 Fragment length distribution of merged reads for mock-up samples (B1, BG1, S1, SG1)

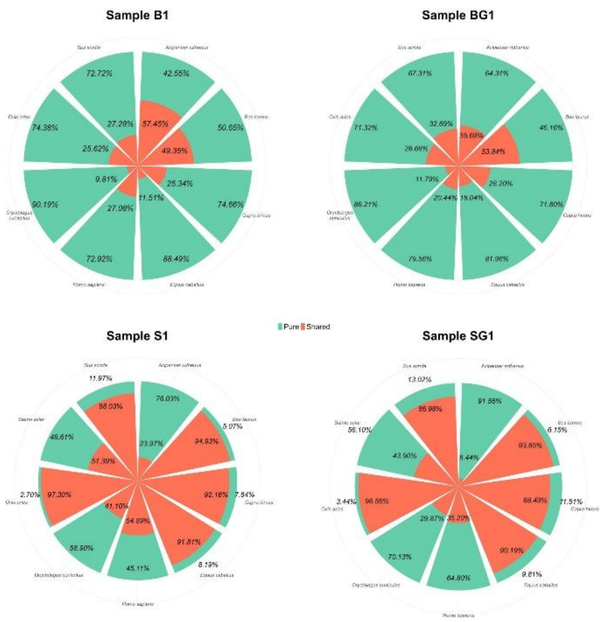

Fig. S4 Evaluation of the potential ubiquity of sequences mapped to the genomes of *Oryctolagus cuniculus, Bos taurus*, *Acipenser ruthenus, Capra hircus*, *Equus caballus*, *Sus scrofa*, *Ovis aries*, *Salmo salar* and *Homo sapiens* for the B1, BG1, S1 and SG1 samples. The percentage of reads unique to the species (pure) is shown in aquamarine green, while the percentage of reads shared among various reference genomes (shared) is displayed in red

Supplementary Tables

Table S1: Bootstrap estimates of low-abundance species (<1%) in sample BA

Species	Species-specific reads (n)	Proportion (%) [95% CI]
Sus scrofa	8,395	0.121 [0.118–0.123]
Equus caballus	8,336	0.120 [0.117–0.122]
Homo sapiens	8,023	0.115 [0.113–0.118]
Acipenser ruthenus	6,882	0.099 [0.097–0.101]

Table S2: Bootstrap estimates of low-abundance species (<1%) in sample CA

Species	Species-specific reads (n)	Proportion (%) [95% CI]
Homo sapiens	8,926	0.504 [0.493–0.514]
Acipenser ruthenus	6,656	0.375 [0.366–0.384]
Oryctolagus cuniculus	4,079	0.230 [0.223–0.237]
Equus caballus	2,331	0.132 [0.126–0.137]

Table S3: Bootstrap estimates of low-abundance species (<1%) in sample SA

Species	Species-specific reads (n)	Proportion (%) [95% CI]
Salmo salar	58,515	0.887 [0.880–0.894]
Ovis aries	13,103	0.199 [0.195–0.202]
Capra hircus	11,411	0.173 [0.170–0.176]
Oryctolagus cuniculus	8,679	0.132 [0.129–0.134]
Sus scrofa	7,427	0.113 [0.110–0.115]
Bos taurus	7,221	0.109 [0.107–0.112]
Homo sapiens	5,800	0.088 [0.086–0.090]
Equus caballus	4,949	0.075 [0.073–0.077]
